# The evolution of health inequalities in the western world: From early origins to modern times

**DOI:** 10.1177/14034948251365433

**Published:** 2026-04-07

**Authors:** Eero Lahelma

**Affiliations:** Department of Public Health, University of Helsinki, Finland

**Keywords:** Socioeconomic inequalities, health inequalities, historical origins, emergence, ubiquity

## Abstract

*Background:*Throughout human history, socioeconomic inequalities in health are shaped by interlinked structural changes, like emerging agriculture and industrialism, demographic and economic transitions, as well as epidemics of infectious diseases, catastrophes, and climate changes. Key questions include when health inequalities emerged, and how they evolved from early communities until modern societies. *Method*: Based on original sources, research literature, reviews, and their critical analysis, this historical review focuses on the evolution of health inequalities, their appearance and advancement. Availability of sources limits the scope to Europe and the western world. *Findings*: Early hunter-gatherer communities were socioeconomically relatively equal, likely lacking major health inequalities. As agriculture emerged in the neolithic time since 10000 BCE, societies became more prosperous and unequal. In medieval societies, with deep socioeconomic inequalities, health inequalities were presumably constrained by infectious diseases and catastrophes hitting populations “democratically”. John Graunt and William Petty showed in Britain novel socioeconomic and health inequalities emerging with early industrialist capitalism. By the “Revolutions of 1848” health inequalities encompassed industrial countries as shown by Rudolf Virchow and Edwin Chadwick. In the Black Report (1980) and present-day research health inequalities appear ubiquitous and often widening. *Conclusions*: The evolution of health inequalities follows both the constancy hypothesis, suggesting omnipresent health inequalities where social inequalities prevail, and the convergence-divergence hypothesis, suggesting variations in health inequalities from prehistory to modern time. A conundrum is, to what extent health inequalities were constrained by epidemics and catastrophes. Currently, health inequalities are a world-wide public issue to be tackled with egalitarian policies.

## Introduction

Understanding differences in population health, disease, and mortality between socioeconomic positions, such as social class, educational attainment, and income group, has made great progress over the last few decades. These differences, generally called socioeconomic inequalities in health, or just health inequalities, contribute to the distribution as well as level of population health and belong to key research issues within public health, social medicine, and medical sociology [1, 2, 3 pp. 90-92].

While most research on health inequalities derives from the post Black Report period since the 1980s [4-6], the topic is not restricted to our times. Nevertheless, historical studies are few and mostly limited to the industrial and post-industrial periods. But when did health inequalities emerge in the western world, and how did they evolve since prehistorical times? To add our understanding, we need better insights into the emergence, coverage, patterning, and evolution of health inequalities from early human communities until modern complex societies.

## Focus and scope

Socioeconomic inequalities in health are commonly characterised as systematic, avoidable, and unfair differences in health outcomes observed between populations and socioeconomic groups, or as a gradient across populations [7]. A characterisation like this aims to encapsulate the current understanding among researchers, practical actors, and policymakers. If unfairness or injustice of health inequalities is being emphasised, the term health inequities has been used [8].

This examination follows the methodological principles of historical review, a special type of literature review. The present literature review follows a semi-systematic approach. It aims to provide an overview of the research area, tracking its development over time, asking relatively broad questions, examining past research, being qualitative in nature and contributing to the state of knowledge ending in a historical overview of health inequalities [9-11]. A historical review examines developments through past events as reported in scholarly contributions. The starting point is typically with the first time a topic, in this case social inequality and health inequality is reported and emerges to conceptual, theoretical and empirical literature. The next step is to trace the evolution of the topics using scholarship through historical periods as material. The purpose is to place phenomena and their research into a historical context, picturing state-of-the-art developments, relevant interpretation models, developmental pathways and perspectives for future scrutiny [10 pp. 24-25].

For the review, original research literature, scholarly commentaries and other relevant textual sources published in scientific fora were searched using Google, Google Scholar, Web of Science and PubMed. Search words “health”, “disease”, “mortality”, “inequalities”, “inequities”, “socioeconomic”, “social class”, “origins”, “history”, “prehistory”, “neolithic revolution”, agriculture, “Middle Ages”, “industrialism” and “industrial revolution” were used. For prehistory the search terms produced a limited number of sources, whereas towards contemporary times the numbers increased exponentially. To find maximally older sources, the above procedures were supplemented by historical articles and books [2-4, 12-19] and “snowballing” literature from their references. The sources retrieved were critically assessed considering their authenticity, relevance, reliability, credibility and consistency, and by whom, when and where they were made and for what purpose [20]. The nature, quality and usefulness of each source for the historical review was checked and, whenever possible, cross-checked using multiple sources. Historical reviews cannot trust on repeated studies like systematic reviews, and a key tool is the historical-critical method or criticism of the sources in assessing the reliability of the textual sources [21].

Health in historical studies, including the present one, refers primarily to mortality, as morbidity data are slim. Determining causes of death varies a lot over historical periods. The magnitude of health inequalities is affected by the overall level of health in a population, with short life expectancy constraining the range of inequalities (12, 16). Factors shaping socioeconomic inequalities in health include gender, ethnic background, age, living arrangements, and neighbourhoods [2-3], but are virtually non-existent in early studies. Even breaking down by sex is exceptional despite variations in life spheres and health [22]. This limitation may cause gender bias in the analysis.

The spatial coverage is largely limited to Europe and the western world due to availability of sources and, even within Europe, a substantial part of studies come from Britain, supplemented by countries like France and Prussia. This limitation directs the focus towards higher income countries with better health and smaller inequalities. Thus, global generalisations are not warranted.

The review proceeds through six broad periods: 1) prehistorical hunter-gatherer communities before 10000 BCE; 2) the neolithic revolution and emergence of agriculture and feudal societies until late Middle Ages in 16^th^ century CE; 3) subsequent early industrialist capitalism and urban development starting the modern era; 4) the breakthrough of industrialism in advanced societies in 18^th^-19^th^ century; 5) the interval between the two 20^th^ century World Wars; and 6) the postwar period until the present day. Two first periods end into a transformation from emerging socioeconomic inequalities to emerging health inequalities. The four final decades witness a development towards strengthening and often widening health inequalities, and finally a massive increase in studies [5, 6].

The aim of this historical review is to trace the emergence of socioeconomic inequalities in health, their evolution and major turning points from early human history to modern times. Key questions include: 1) Has *socioeconomic* inequality always been there, or when and why did it emerge? 2) Has *health* inequality always been there, or when and why did it emerge? 3) What circumstances have shaped health inequalities over their evolution? [16, 23-26]

Two contrasting hypotheses have been proposed, predicting the development of health inequalities [27]. First, the constancy hypothesis suggests that health inequalities are inherent, perpetual and omnipresent in human life across time, place, level of living and state of health. The omnipresence of health inequalities is due to general susceptibility, historical inevitability and fundamental causes leading to differential relative disease risks between hierarchical socioeconomic positions [28-31]. Second, the convergence-divergence hypothesis suggests that health inequalities are unlikely to be historically omnipresent but have been temporarily and recurrently counteracted. Thus, high maternal and child mortality, fatal infectious diseases, wars and catastrophes, as well as climate changes, may have constrained the presence and strength of health inequalities. These hardships have hit largely “democratically” populations and subgroups from elites to common people until the dawn of modern era [26, 27, 32].

Once appearing, health inequalities likely show divergence and convergence over time and place. This is due to variations in economic developments and hardships as well as in individual characteristics, like gender and age, across socioeconomic positions [12, 16, 26, 27, 32-34].

## From early socioeconomic equality to the emergence of inequality

The review starts from the early human history, referring briefly to a long period from ca. 300000 BCE to 10000 BCE, when people lived in small hunter-gatherer communities comprising of 10-50 members. Subsistence was based on hand-to-mouth existence and the distribution of commodities basically on need. The early communities were presumably relatively equal, lacking steep and established social structural hierarchies (16 p. 76, 19 p. 144, 23 p. 46, 34 p. 30). Life chances were limited as up to two thirds of deaths could be due to maternal and child mortality as well as epidemics of infectious diseases such as tuberculosis, malaria, influenza and smallpox. People were likely relatively equally hit by these hardships, constraining time and again the magnitude of health inequalities but at a short life expectancy of 20-40 years [12, 26, 27, 35].

The neolithic revolution from 10000 years BCE saw the dawn of agriculture, with people settling down and populations growing larger. The transition from foraging communities to non-nomadic societies was a multifaceted process, which may have had varying impacts on morbidity and mortality in different populations. Paleodemographic evidence suggests that agriculture contributed to social inequalities and led to greater morbidity e.g. due to dental problems and bone lesions. Once agricultural economies were established, the overall population started to grow. These transitions were followed by changes in living conditions, ways of life and cultures. Novel civilisations based on agriculture emerged first in Mesopotamia, and subsequently broader areas of Afro-Eurasia [16 pp. 78-80, 36-37].

Transition to agricultural economies started to yield surplus, no longer distributed according to need but power. With increasing productivity, agricultural societies became more prosperous, but also more unequal. Gerhard Lenski [23 pp. 44-46] argues that unequal distribution of power and resources, i.e. social stratification, is an inherent characteristic in human communities (at least) since the emergence of agriculture. To encourage innovation and produce surplus, agricultural societies needed some degree of social inequality. This contributed the communities and societies to add their productivity. Since the neolithic agricultural revolution, social inequalities and economic growth have gone hand in hand. The key counterforces for the increase in the early inequality were paradoxically violence, wars, catastrophes and fatal epidemics which then hit population subgroups from the highest to the lowest ones [12, 26, 27, 34 pp. 6, 30].

Archaeological data with robust gini coefficients and regression analyses suggest that post-neolithic socioeconomic inequalities tended to widen as the stage and intensity of agriculture, sociopolitical development and urbanisation proceeded. The magnitude of these inequalities showed further variations between areas and societies. Thus, economic inequalities in post-neolithic Mesopotamia and elsewhere in Afro-Eurasia were likely larger than in the New World [37].

It is reasonable to assume that the economic inequalities gave rise to early social inequalities and further to health inequalities, but firm evidence is lacking. According to sporadic mentions in the literature there are signs of possible health inequalities in some ancient societies like Mesopotamia, Greece and Rome [16 p. 82, 38, 39].

## Socioeconomic inequality and health inequality since the Middle Ages

During the period called the Middle Ages, i.e. about 5^th^-16^th^ century CE, socioeconomic inequalities in Europe and elsewhere followed the feudal order. The ruler and the supporting noble and clerical peerage constituted a small and relatively homogeneous elite or upper class, whereas peasants and other common people constituted a vast and amorphous underclass [26, 33, 40, 41]. The medieval underclass also included serfs and slaves, suffering from particularly deep inequalities and high mortality [12, 42].

Towards the late Middle Ages, the elite stuck to their privileges, this further deepening socioeconomic inequalities and deprivation among common people [16, 40]. In communities and societies, socioeconomic inequalities and health inequalities were both shaped by complex influences. These include steeply unequal distributions of power and resources between the elite and the subgroups of common people. As for earlier periods, highly virulent and fatal epidemics as well as catastrophes, wars and violence continued as major killers throughout medieval populations, keeping life expectancy at a previous low level [12, 26, 27, 32].

Questions can be raised, what might be the on-balance effects of socioeconomic inequalities and the major killers on health inequalities [25, 26, 40, 43 pp. 8-12]. It has been argued that health inequalities remained minor, in some cases possibly even non-existent until about mid seventeenth century [12 p. 36, 27 p. 346, 43 p. 11]. This counterintuitive socioeconomic patterning of health has been attributed to the major killers penetrating communities and their subgroups, resulting to wide-ranging mortality for which prevention and treatments were practically unavailable. The dramatic consequences are exemplified by the peak of Black Death in 1348-1349 in England, after which the population almost halved [26].

The elites were though able to strive for a longer life with their access to material resources, better nutrition and shelter, but even they lacked efficient means to avoid the fatal epidemics and other catastrophes, leading to so-called “peerage paradox” [12, 16 pp. 82-83, 26-29, 43-45]. While we can assume that in medieval societies relative health inequalities may have been constrained by counteracting factors like the major killers hitting population subgroups throughout, this is likely to have been mostly temporary and recurrent. The deep structures of inequalities in people’s life and their health remained basically immutable.

In medieval societies, life expectancy hit constantly the rock bottom of 20-40 years, this being a further condition constraining the variation between population groups [12, 16, 26-27, 46]. Overall, studies on Middle Ages are few and based on limited data sources and crude socioeconomic divisions between elites and common people.

An exceptional study focusing on the early 17^th^ century population of Geneva [47] used data allowing an almost modern classification to three balanced occupational classes, age groups and two genders. In the topmost class, life expectancy at birth was 37 years for women and 35 for men, in the intermediate class it was 27 and 24 years, and in the lowest class 21 and 18 years, respectively. The inequalities were graded and large, largest among newborns, but visible throughout age groups, whereas gender differences were minor. While these findings suggest clear health inequalities already before industrialism, they may reflect the particular population and class structure of a relatively affluent protestant city state of Geneva [48].

## Towards modern era: the pioneering work of Graunt and Petty

Since the 16^th^-18^th^ centuries, Britain and other advancing European societies took steps towards industrial capitalism, followed by demographic transitions and novel socioeconomic inequalities. Mass-scale urbanisation and industrialisation exposed the expanding working class to heavy working conditions and over-crowded housing, with adverse class-related health consequences. Among the large urban-industrial proletariat, mortality remained high, and the previously short life expectancy tended to shorten further. Also, among the British elite, accounting for only one percent of the population, life expectancy remained short, but still longer than among the proletariat [12, 16 pp. 82-83, 40, 41].

In Britain, early ideas of mercantilism and enlightenment were catalysts for social, economic, and political development marking the dawn of modern era. Importantly, population health was understood as a resource for the wealth of nations as in the work of John Graunt (1620-1674) and William Petty (1623-1687), two ground-breaking population health researchers [49-52].

Graunt’s study, *Natural and Political Observations Mentioned in a following Index, and Made Upon the Bills of Mortality*, from 1666 [5] is a unique statistical analysis of changes in the 17^th^-18^th^ century mortality across subgroups, enabled by new data source, London Bills of Mortality, initiated in 1532. Graunt invented innovative concepts and methods, such as statistical association, excess mortality and life expectancy and, as today, his key variables were sex, age, place, religion, and socioeconomic position [49-52, 53 p. 99, 54]. These were important tools in the early analysis of health inequalities.

Graunt found that common causes of death among men were violence, accidents, and dangerous work and, among women, obstetric and puerperal troubles. Poor housing, difficulties in the family, loneliness and unhealthy habits provided further backgrounds for high and unequal death rates [53 pp. 62-67].

Over-crowded and poverty-stricken neighbourhoods of London suffered from particularly high mortality, urban penalty”. Graunt’s analyses confirmed the association between adverse living conditions and mortality. Polluted and unhealthy air varied across London neighbourhoods, and this was seen as spatial inequalities in mortality. Forty percent of native Londoners reached the age of 16, whereas the proportion among the elite was almost double [43, 49, 53 pp. 91-99].

Petty collaborated with Graunt and continued his work, including utilisation of research evidence in policies and health care [54]. He adopted a modern egalitarian view implying that deprived people should be integrated to society by supporting the poor, sick and unemployed, and by offering education and health care for all [52, 55, 56, 57 pp. 37-49]. The work of these two pioneers provided novel research methods and demonstrated the presence of health inequalities as well as their background factors under emerging industrial capitalism. They were well ahead of their own time and it took almost 200 years before they had successors [49-51].

## Revolutions of 1848: breakthrough in research and action

With expanding industrialism, the feudal estate order eroded and a novel stratified socioeconomic structure, composed of upper ruling class, intermediate classes and lower working class became the dominant model. From the mid-18^th^ century on, life expectancy lengthened little by little in many European countries, with diminishing between-country disparities. The lengthening was faster among the upper classes, strengthening the class inequalities in health. With the 19^th^ century industrial class structure, within-country health inequalities were exacerbated [16 pp. 29, 94-95, 44, 46, 58, 59].

New ideologies, liberalism, nationalism, and socialism gave impetus to social and political movements, unrest and contradictions. ”Das tolle Jahr” or “Revolutions of 1848” became symbolic for the whole epoch, and Rudolf Virchow (1821-1902), a medical doctor active in Prussia and Bavaria, was its key promoter of public health. His paradigmatic study of typhoid epidemic in Silesia in 1848 showed that a tenth of the population contracted the disease and a fifth of the diseased died, disproportionately the poor [60]. Virchow’s reform programme included better working conditions and he suggested education and health care for all like Petty had done 200 years earlier [61, 62].

In Britain, Edwin Chadwick (1800-1890), William Farr (1807-1883), and Friedrich Engels (1820-1895) and, in France, Louis-René Villermé (1782-1863) continued studies on class inequalities in health [63-65]. Engels reported that in Manchester mortality at the poorest housing level was 78 percent higher than at the highest level [65 p. 134]. Chadwick and Farr contributed to British statistical authority, which initiated regular reporting on health inequalities in 1837 [66-68].

Chadwick analysed living conditions and health among the urban and rural working class in his *Report on the Sanitary Conditions of the Labouring Population of Great Britain* from 1842 [68, 69]. In Liverpool - then the “unhealthiest city in England” [63] - the average age at death was 55 years in the highest class, but only 25 years in the lowest class. In the rural county of Rutland, the corresponding figures were 52 and 38 years [62 p. 164]. The Lancet also noted the high mortality and the large class inequalities among the urban population [70 p. 660].

Awareness of health inequalities spread throughout Europe. Even in a small and peripheric country of Finland, class inequalities culminated in late 19^th^ century, and several studies analysed inequalities in morbidity and mortality [71]. For example, Carl Qvist [72 p. 43], concluded in 1872 that “only in exceptional cases have the better-off classes fallen victim of pernicious cholera as often as the lower classes”.

## Progress in empirical analysis

Social class remained a vague and poorly operationalised concept in research. British scholar T.H.C. Stevenson (1870-1932) formulated a novel social class classification suitable for studies made under the established class inequalities of industrialised societies [73]. His classification was not based on class theoreticians, i.e. Karl Marx, who focused on people’s relationships to production, ownership, and material resources, or Max Weber, who focused on markets, status, and consumption. Instead, Stevenson’s approach was practical and ”pseudo-analytical” [74], including occupation as a primary criterium for class position and, skill level, affluence/poverty, hygiene, lifestyles, education, and class cultures as secondary criteria, as many subsequent classifications did. Following the ways of time, class position of a family was determined by the (male) “head of household” [14, 75, 76].

Stevenson’s elaborated classification was purely hierarchical and followed an ordinal scale: 1) upper and middle classes; 2) intermediate classes between 1 and 3; 3) skilled workmen; 4) intermediate classes between 3 and 5; and 5) unskilled labourers [75]. The classification showed good discriminatory power and consistency across studies. It was used in British official statistics up to 1980s and even outside Britain [77].

In his own study, Stevenson showed that both total and child mortality were consistently higher in lower classes [75]. Richard Titmuss (1907-1973), applying Stevenson’s classification, found that child mortality in Britain declined in each class from 1910s to 1930s, but faster in the uppermost classes, resulting to widening mortality inequalities [14, 78]. This mechanism of change has turned out common for health inequalities.

Outside Britain, the study of health inequalities rested largely on individual scholars. In the USA, the work of Edgar Sydenstricker (1881-1936) was important [79]. Studying the Spanish flu pandemic, bursting out in 1918 and continuing during WWI, he reported lower class overrepresentation among the deceased [80].

## Dormant health inequalities and revival

Reconstruction period after WWII contributed to prosperity in many countries, with life expectancy lengthening globally and converging among affluent countries [46, 59]. Class inequalities remained but health inequalities were temporarily neglected, which may have given rise to their presumed decline. Antonovsky [12] reckoned that in the 1960s health inequalities were smaller than ever before, and Charles Kadushin (1932-2022) presumed that, with declining absolute poverty and universal health care, health inequalities most probably no longer existed in western countries [81]. Nevertheless, health inequalities had survived and even widened as accumulating evidence from the USA and other countries confirmed [82-84].

In Britain, the government’s question was why the national health care NHS had been unable to abolish health inequalities despite its 30 years of operation. To find the answer the British Labour government set up a commission in 1977. Its report summarised the prior studies and the commission’s own scrutiny on Britain, led by Peter Townsend (1928-2009) [85]. The outcome, *Inequalities in Health - The Black Report* [4], marked a dramatic increase in the awareness and studies of health inequalities in the early 1980s. Published as a book it became likely the broadest ever spread and most cited public health study [4 pp. 16-18, 85].

As summarised by the Black Report morbidity and mortality were consistently higher in lower classes in Britain and elsewhere. The inequalities were seen from childhood to late adulthood and concerned women and men as well as most diseases and causes of death [4 pp. 51-64, 85]. Health care use was unequal as well, but the idea to attribute health inequalities simply to shortage of health care was discarded.

The Black Report gave an impetus to a series of national and international reviews of health inequalities [15, 86-88]. Bibliometric analyses show that before 1980s studies were infrequent but increased soon exponentially, amounting to 70000 by late 2010s. Further countries like the USA, the Netherlands, and the Nordic countries showed an active research input on health inequalities [5, 6, 19 p. 6].

Towards the present day, several interlinked processes have been identified to contribute to socioeconomic inequalities and further health inequalities in European and other western countries [89]. Since the 1970s, neoliberalist ideology has emphasised restructuring of the economy, free markets, privatisation, smaller state interventions and flexible labour markets, thus creating an atmosphere for stronger socioeconomic and health inequalities [90]. Austerity policies, recessions and the 2008 financial crisis have additionally contributed to poorer living conditions and wider relative inequalities in many countries. The national policy responses vary from promoting or counteracting austerity [19, 91]. The COVID-19 pandemic from 2020 on further exacerbated endemic health inequalities and resulted in a syndemic pandemic, with higher mortality and morbidity rates among the socially disadvantaged. These developments have continued to add to the existing burden of health inequalities across countries [92].

## “Nordic paradox”

While evidence on health inequalities in single countries accumulated, a picture on the international variation was hard to reach from country-specific studies only. An effort to fill this gap was a comparative study on socioeconomic inequalities in health across European countries, led by Johan Mackenbach from Erasmus University Rotterdam, the Netherlands [19].

The first phase of the study in 1980s-90s covered eleven western European countries, and its main report was published in the Lancet [93]. All countries showed clear and consistent relative inequalities in morbidity and mortality. Surprisingly, health inequalities in Sweden or other Nordic welfare states were not smaller than elsewhere. Rather, relative inequalities were larger than in southern European countries with their smaller emphasis on equality [94, 95].

Next, the comparisons were extended to 40 European countries. In Eastern central European countries, like Poland, Hungary and Lithuania, relative inequalities in mortality were largest and showed largest widening from 1970s to 2010s simultaneously with major social transformations in these countries. In central and southern Europe, widening was minimal or non-existent [19 pp. 23-27, 33-36]. Also, absolute inequalities in mortality widened in Eastern central European countries, whereas in western Europe, including the Nordic countries, they remained or even narrowed [19 pp. 23-27, 96].

In the Nordic countries, large relative inequalities in mortality have persisted and absolute inequalities among women are at the highest western European level. Promoting egalitarian health and welfare policies has not safeguarded smaller health inequalities in the Nordic countries compared to their western and southern European counterparts [19 pp. 25, 145-146, 163-166, 97, 98]. This patterning has given rise to name the phenomenon the “Nordic paradox” or “welfare state paradox” [19 pp. 133-134, 97].

Life expectancy in the Nordic countries is at high European level, although not at the absolute top [99]. To better understand the Nordic paradox, other circumstances than policies only should be considered. For example, income inequalities are seen equally in the Nordic countries, and poverty and marginalisation are neither unknown. Furthermore, inequalities in lifestyles, like smoking, drinking, nutrition and exercise contribute to Nordic health inequalities [100].

In all Nordic countries, life expectancy has increased steadily, but upper classes tend to benefit most from this increase upholding the paradoxical development. For example, among Finnish low-income men life expectancy stagnated in the 1990s and again in the 2010s. Trends like these contribute to widening relative inequalities in life expectancy, as discovered by Richard Titmuss one hundred years ago [19 pp. 172-174, 59, 78, 101].

## Discussion

### Overall evolution

From early human communities to modern complex societies, social and health inequalities in the western world are inherent and perpetual across time, place, and level of health. There are variations, on the one hand, by economic and social structural conditions and transformations and, on the other hand, by maternal and child mortality, infectious diseases, catastrophes and climate changes [26, 27, 32].

First, early hunter-gatherer communities were relatively equal, with likely limited inequalities also in the face of death and disease [16 pp. 78-82, 26].

Second, after the emergence of agriculture during the neolithic revolution since 10000 BCE, unequal socioeconomic structures were gradually developed [23 pp. 44-46]. Feudalist social order in the Middle Ages cemented the cleavage between elites and common people. While socioeconomic inequalities existed, health inequalities were likely temporarily and recurrently constrained by major killers, like fatal epidemics and catastrophes hitting not only common people but also elites [12, 16 pp. 78-82, 26-27, 32]. Even so, it remains a key conundrum, needing further scrutiny, when, where and to what extent health inequalities were constrained under the socioeconomic inequalities as well as life-threatening health hazards until late the Middle Ages.

Third, during the period of emerging modern era and industrialism, John Graunt and William Petty demonstrated rising socioeconomic inequalities in mortality in Britain. By the “Revolutions of 1848”, class inequalities in health were seen in societies undergoing the breakthrough of industrial capitalism [62-64].

Fourth, in the 20^th^ century, health inequalities appear ubiquitous, although occasionally neglected. The Black Report [4] and the following boom of research re-confirmed wide-spread health inequalities [5, 6, 13], warranting an invariance: the lower the socioeconomic position, the poorer the health [18 pp. 23-24, 102, 103]. Subsequently, neoliberalist ideologies, austerity policies and the COVID-19 pandemic added to the existing burden of health inequalities [92].

The boom also gave an incentive to scientific and policy-oriented discussions. One debate is about whether health inequalities are narrowing, widening, or remaining constant [12, 16 p. 82, 19, 22, 27]. In many countries, relative inequalities have widened or remained, with no evidence of narrowing, whereas the trends for absolute inequalities are more varied, showing more stability and even narrowing in some cases [19, 59, 97, 101, 104]. Another debate is about interventions and policies aiming at smaller health inequalities. Programmes for reducing health inequalities have been launched at the international and national level. An ambitious global effort was the WHO Commission on Social Determinants of Health in 2005-2008 (15) highlighting equality of living conditions as well as material and other resources as a basis for equity in health. Another ambitious effort was the British 1997-2010 strategy to reduce health inequalities, with narrowing mortality inequalities among elderly people but limited overall effects [105, 106]. The task has turned out a tricky one, but the efforts have not been wasted as without them inequalities might be even larger. The same concerns the Nordic paradox as universal social policies have likely buffered against larger health inequalities [19 pp. 133-134, 95].

Towards the present day, many Western countries have faced adverse social and economic developments. Governments have increasingly followed neoliberalist ideologies and resorted to austerity policies, with cuts in health and welfare spending [91]. Lower classes suffer disproportionately from unemployment, poor and unequal living conditions, contributing to pressures towards widening future health inequalities [19 p. 182].

### Limitations

A historical review with a very long timeline is susceptible to limitations, uncertainties and sources of error, which may distort the conclusions drawn. Firstly, data for the early phases are limited and original sources few. Secondly, historical health data cover almost exclusively mortality. However, people often suffer and die from similar conditions. Thirdly, past mortality and morbidity data differ from the current data and constructs, such as diagnoses and causes of death. Fourthly, socioeconomic structures, class concepts and classifications vary over time and place. Fifthly, serfdom and slavery represent extreme deprivation but have been neglected in the historical examination of health inequalities. Sixthly, key factors shaping health inequalities, in particular, gender and ethnicity are practically lacking in data sources and studies reviewed. Seventhly, the significance of folk medicine and early medical practices is unknown. Eighthly, evidence is particularly incomplete on changes in health inequalities during the transitions from the neolithic to the medieval and further to early modern societies. Finally, the spatial coverage of the preceding review is limited, and includes at best Europe and the western world. However, health inequalities elsewhere need not follow western pathways and unilinear trends. Consequently, global generalisations are not warranted.

## Conclusion

Reviewing the evolution of socioeconomic inequalities in health shows major variations from the early origins until present. According to the constancy hypothesis, health inequalities, largely similar to modern ones, would be inherent in human life and seen across time, place and level of health [30, 31].

However, among the prehistorical hunter-gatherer communities major socioeconomic inequalities and substantial health inequalities have likely been limited. The emergence of neolithic agricultural revolution was followed by unequal power structures and socioeconomic inequalities. It remains unclear to what extent this was accompanied by health inequalities. If health inequalities were constrained this would be attributed, in the first place, to the major killers including maternal and child mortality as well as fatal infectious epidemics and catastrophes hitting populations and subgroups “democratically” until late Middle Ages [12, 26, 27, 32]

Towards the modern era, health inequalities emerged simultaneously with novel socioeconomic class structures. With the breakthrough of industrial capitalism, health inequalities strengthened and have shown persistence ever since. While much uncertainty remains, the evolution of health inequalities is likely better consistent with the convergence-divergence hypothesis, suggesting variations from prehistorical periods to modern era. In turn, the consistency hypothesis, as based on the notion of fundamental causes, is better suitable for periods after the emergence of health inequalities [30, 31].

These conclusions derive from the western developments, whereas the history of non-western health inequalities remains practically unreported. Historical reviews are justified not only as they provide perspectives for past trends, but equally as they add our understanding of the present health inequalities, their evolution, spatial coverage and driving forces.
